# Amelioration of lipopolysaccharide-induced liver injury by aqueous rooibos (*Aspalathus linearis*) extract via inhibition of pro-inflammatory cytokines and oxidative stress

**DOI:** 10.1186/1472-6882-14-392

**Published:** 2014-10-13

**Authors:** Olawale Razaq Ajuwon, Oluwafemi Omoniyi Oguntibeju, Jeanine Lucasta Marnewick

**Affiliations:** Oxidative Stress Research Centre, Institute of Biomedical and Microbial Biotechnology, Faculty of Health and Wellness Sciences, Cape Peninsula University of Technology, PO Box 1906, Bellville, 7535 South Africa; Redox Laboratory, Department of Human Biology, Faculty of Health Sciences, University of Cape Town, Anzio Road, Observatory, 7925 Cape Town, South Africa

**Keywords:** *Aspalathus linearis*, Anti-inflammatory, Cytokine, Hepatotoxicity, Lipopolysaccharide, Oxidative stress

## Abstract

**Background:**

Acute liver injury occur after intraperitoneal administration of lipopolysaccharide (LPS). Oxidative stress and release of pro-inflammatory cytokines are both implicated in the pathogenesis of LPS-induced acute liver injury. This study investigated the ameliorative effect of fermented rooibos (*Aspalathus linearis*) extract on LPS-induced acute liver injury.

**Method:**

Major phenolic compounds in the fermented rooibos extract by HPLC-DAD, as well as the *in vitro* antioxidant capacity were quantified before the start of the experiment. Male Wistar rats were randomized into 4 groups (n = 10 per group) and given either water or fermented rooibos extract for 4 weeks before LPS injection. Hepatic function markers, including aminotransferases and lactate dehydrogenase, lipid peroxidation markers, antioxidant enzymes, glutathione redox status, as well as cytokine levels were monitored in the rats.

**Results:**

Injection of LPS significantly increased serum levels of alanine aminotransferase (ALT), aspartate aminotransferase (AST) and lactate dehydrogenase (LDH). Oxidative stress, evidenced by increased thiobarbituric acid reactive substances (TBARS) measured as malondialdehyde (MDA) in plasma and liver, and decreased glutathione redox status (GSH: GSSG ratio) in whole blood and liver was induced in LPS-challenged rats. Furthermore, hepatic levels of pro-inflammatory response markers TNF-α, IL-1β and IL-6 were increased significantly. Pre-feeding the fermented rooibos extract for 4 weeks decreased LPS-induced elevated levels of serum AST and LDH (significantly, p < 0.05) as well as ALT marginally. Consuming rooibos caused an attenuation of the observed increase in plasma and hepatic MDA, decrease in whole blood and liver GSH:GSSG ratio, as well as the changes noted in various antioxidant enzymes. The elevation in TNF-α and IL-6 was significantly suppressed, indicating an inhibition of the induced inflammatory response by rooibos.

**Conclusion:**

Overall, our data showed that aqueous rooibos extract attenuated LPS-induced liver injury possibly by modulating oxidative stress and suppressing pro-inflammatory cytokines formation.

## Background

Lipopolysaccharide (LPS), an endotoxin, is a major glycolipid component of the outer cell wall of gram-negative bacteria, made up of a polysaccharide O-chain and a biologically active lipid-A moiety, embedded within the bacterial membrane [1]. Endotoxemia-induced toxicity is characterized by injury to various organs, including liver, kidney and the brain, and it has been implicated as a contributing factor to bacterial infection resulting in sepsis, which is one of the major causes of morbidity and mortality in intensive care units [2]. Most of the toxicities observed in LPS-induced injury in the liver and systemic circulation has been attributed to toxic mediators produced by activated macrophages, including cytokines, such as tumor necrosis factor-α (TNF-α), interleukins (IL-1, IL-6, IL-8, and IL-12), other pro-inflammatory molecules, including platelet-activating factor, prostaglandins, as well as reactive oxygen and nitrogen species (RONS), such as nitric oxide (NO) and superoxide radical [3]. The systemic inflammatory response triggered in the host by LPS is characterized by fever, leucocytosis, thrombocytopenia, changed metabolic responses and redox status impairment [2]. The consequences of impaired intracellular redox balance includes the generation of excessive RONS, induction of lipid peroxidation, DNA and protein damage, depletion of intracellular stores of endogenous antioxidants and inhibition of antioxidant enzymes [4].

The involvement of oxidative stress in injury associated with LPS suggests that dietary antioxidants may enhance the efficacy of treatment protocols designed to mitigate LPS-induced endotoxemia. Medicinal plants, fruits, vegetables, spices and teas are drawing a lot of attention because of their demonstrated health benefits, with scientific evidence demonstrating that phytochemicals in fruits, vegetables, spices and teas possess a high number of protective biological properties, including antioxidant, anti-inflammatory and other beneficial effects [5].

Rooibos (*Aspalathus linearis*) (Brum f) Dahlg. (Family Fabaceae; Tribe Crotolarieae) is a shrubby legume that is indigenous to the mountainous area of Clanwilliam in the Western Cape Province of South Africa. Traditionally, it is used to make a herbal beverage that is naturally caffeine-free, low in tannin and rich in unique polyphenolic antioxidants [6]. Due to its rich content of different compounds, some unique, with antioxidant and other health properties, rooibos is gaining more attention worldwide because of its potential for clinical uses. Aqueous extracts of rooibos have been shown to possess antioxidant activities *in vitro* [7]. *In vivo* evidence has shown that aqueous rooibos extracts are able to modulate oxidative stress by inhibiting lipid peroxidation and augmenting the glutathione redox status in rat sperm [8], rat liver [9] and in humans with an occupational risk [10] and at the risk of developing cardiovascular diseases [11]. Immunomodulatory effects of rooibos have been previously reported both *in vitro* and *in vivo* [12, 13], while a recent study also showed that rooibos and two of its flavonoids (luteolin and quercetin) were able to reduce the secretion of pro-inflammatory cytokine, IL-6 and TNF-α using a LPS-stimulated macrophage model [14]. However, the evaluation of the *in vivo* anti-inflammatory properties of rooibos remains to be sufficiently studied. Rooibos is most often consumed as a herbal tea and therefore an aqueous (hot-water) extract of fermented rooibos leaves and stems at a similar concentration as consumed by humans traditionally were used in this study to investigate its effectiveness in protecting against LPS-induced hepatic injury in Wistar rats.

## Methods

### Preparation of the aqueous rooibos extract

Fermented rooibos (superior grade) plant material was a generous gift from Rooibos Limited (Mr Arend Redelinghuys, Clanwilliam, South Africa). An aqueous rooibos extract (2%, w/v), was prepared as reported previously [15] by the addition of freshly boiled tap water to tea leaves and stem at a concentration of 2 g/100 mL. The mixture was allowed to stand at room temperature for 30 minutes with constant stirring, filtered afterwards and dispensed into water bottles. The extract was fed to the rats *ad libitum,* with fresh extract prepared every second day.

### Phenolic content and total antioxidant capacity of aqueous rooibos extract

The soluble solids content of the rooibos extract was determined gravimetrically as previously reported [15]. The total polyphenol content of the aqueous rooibos extract was determined using the Folin Ciocalteu’s phenol reagent according to the method described by Singleton *et al* [16] and results expressed as mg gallic acid equivalents/mg soluble solids. The flavanol content of the aqueous rooibos extract was determined colorimetrically at 640 nm using *p*-dimethylaminocinnamaldehyde (DMACA) according to the method of Treutter [17]. Results were expressed as mg catechin standard equivalents/mg soluble solids while the flavonol/flavones content was determined spectrophotometrically at 360 nm and results expressed as mg quercetin standard equivalents/mg soluble solids [18]. The total antioxidant capacity of the rooibos extract was assessed by measuring the oxygen radical absorbance capacity (ORAC), trolox equivalent antioxidant capacity (TEAC) and ferric ion reducing antioxidant power (FRAP) according to methods described by Ou *et al*. [19], Re *et al*. [20] and Benzie and Strain [21] respectively with some modifications as reported previously [9].

### HPLC analyses of aqueous rooibos extract

Quantification of the major phenolic compounds in the aqueous rooibos extract was performed by high performance liquid chromatography (HPLC) on an Agilent 1200 series instrument (Agilent Technologies, Waldbron, Germany) equipped with an in-line degasser, quaternary pump, autosampler and a diode array and multiple wavelength detector, using a 5 μm YMC-Pack Pro C18 (150 mm × 4.6 mm i.d.) column for separation, with acquisition set at 287 nm for aspalathin and 360 nm for other components. The mobile phases consisted of water containing 300 μL/L trifluoroacetic acid (A) and methanol containing 300 μL/L trifluoroacetic acid (B). The gradient elution started at 95% A, changing to 75% A after 5 min and to 20% A after 25 min and back to 95% A after 28 min. The flow rate was set at 0.8 mL/min, the injection volume was 20 μL and the column temperature was set at 23°C [22]. Peaks were identified based on the retention time of the standards and confirmed by comparison of the wavelength scan spectra (set between 210 nm and 400 nm).

### Animal treatment and experimental design

Forty pathogen-free, male Wistar rats weighing 287 ± 11 g were obtained from the Animal Unit of Stellenbosch University (Tygerberg Campus, South Africa). The rats were housed individually in stainless steel wired top and bottom cages fitted with polypropylene houses in an experimental animal holding facility maintained at a temperature of between 21-24°C, with a 12 h light dark cycle. The rats were fed standard rat chow (SRC) *ad libitum* and had free access to tap water or the aqueous rooibos extract. After acclimatization for 1 week, the rats were randomized into four groups of 10 animals each, and treated for 28 days as follows: groups I (control) and II (LPS), were fed SRC and received tap water as the sole source of drinking fluid, while groups III (rooibos) and IV (rooibos + LPS) were fed SRC and received the aqueous rooibos extract (2%, w/v) as the sole source of drinking fluid. On the 27^th^ day of the experiment, the rats were injected with either 0.1 mL of PBS vehicle (groups I and III) or 0.1 mL of LPS (*Escherichia coli* serotype 0111:B4, 0.5 mg/kg bw, i.p.) for groups II and IV to induce endotoxemia and liver injury [23]. Animals used in the study received humane care in accordance with the Principle of Laboratory Animal Care of the National Medical Research Council and the Guide for the Care and Use of Laboratory Animals of the National Academy of Sciences (National Institute of Health Publication no. 80-23, revised 1978). The study protocol was approved by the Cape Peninsula University of Technology’s Faculty of Health and Wellness Sciences Research Ethics Committee (Ethics Certificate no: CPUT/HAS-REC 2010/A003). The general conditions of the rats were monitored daily throughout the study and body weights recorded weekly and at sacrifice. Fluid intake was monitored at intervals of 2 days for the duration of the study period. At the end of the experimental period, fasted animals in all the groups were sacrificed 16 hours after the last LPS injection under sodium pentobarbital anesthesia (0.15 ml/100 g body weight, i.p.). Approximately 8 ml of blood was collected via the abdominal aorta and this was aliquoted into tubes with or without EDTA to obtain plasma or serum, respectively. The liver was excised, washed twice with ice-cold PBS (10 mM phosphate buffered saline pH 7.2) to remove residual blood, blotted to dry, weighed and immediately snap frozen in liquid nitrogen and stored at -80°C for biochemical analyses.

### Preparation of liver homogenate

Liver tissue was minced and homogenized (10%, w/v) in 50 mM NaH_2_PO_4_ buffer containing 1 mM EDTA and 0.5% Triton-X (pH 7.5) on ice. The homogenate was centrifuged at 10 000 g for 10 minutes at 4°C. The resulting supernatant was collected and stored at -80°C until used for biochemical assays. Protein contents of samples (erythrocyte and liver homogenate) were determined using a BCA protein assay kit (Pierce, Illinois, USA).

### Antioxidant capacity of plasma and liver homogenate

To avoid protein interference in the antioxidant capacity assays, sub-samples of plasma and liver homogenates were precipitated with 0.5 M perchloric acid (1:1, v/v) and centrifuged at 10 000 g for 10 min at 4°C. Supernatants were collected as protein free fractions [24]. Plasma total polyphenol content, as well as the ORAC (plasma and liver) were carried out according to the method of Singleton *et al*. [16] and Ou *et al*. [19], respectively.

### Liver function tests

Serum ALT, AST and LDH were determined using standard diagnostic kits supplied by Medica Corporation, (Bedford, Mass., USA), on a Medica EasyRA automated clinical chemistry analyzer (Medica Corporation Bedford, Mass., USA).

### Lipid peroxidation, antioxidant enzymes activities and glutathione status determination

Lipid peroxidation was estimated by measuring conjugated dienes (CD) and malondialdehyde (MDA). Plasma and liver MDA were assayed as MDA-TBA adducts using HPLC with a UV-visible detector according to a method of Khoschsorur *et al*. [25]. Results were expressed as μmole/L or μmole/g tissue in plasma and liver respectively. Conjugated dienes were estimated according to the method of Recknagel & Glende [26] and results expressed as nmole/L or nmole/g tissue in plasma and liver respectively.

Antioxidant enzyme activities were determined in the erythrocytes and liver homogenates. Catalase (CAT) activity was determined according to the method described by Aebi [27] in which the rate of decomposition of hydrogen peroxide was measured at 240 nm. The activity of catalase was calculated using a molar extinction coefficient of 43.6 M^-1^ cm^-1^ and results expressed as μmole H_2_O_2_ consumed/min/μg protein. The activity of superoxide dismutase (SOD) was determined according to the method of Crosti *et al*. [28] and activity expressed as U/mg protein. Glutathione peroxidase (GPx) activity was determined according to the method described by Ellerby & Bredesen [29]. The activity of GPx was calculated using the extinction coefficient of 6.22 mM^-1^ cm^-1^ and results expressed as nmole NADPH oxidized per min per μg protein. Glutathione reductase (GR) was assessed by the method of Staal *et al*. [30] and result expressed as μmole NADPH oxidized per min per μg protein using the extinction coefficient of 6.22 mM^-1^ cm^-1^.

Hepatic and whole blood glutathione redox status was measured using a Bioxytech GSH/GSSG-412™ kit (OxisResearch™, Portland, USA). This method was based on a method initially developed by Tietze [31] in which Ellman’s reagent (5,5’-dithiobis-2-nitrobenzoic acid, DTNB) reacts with GSH to form a product spectrophotometrically detected at 412 nm. Briefly, aliquots of whole blood without (total glutathione) or with 3 mM freshly prepared 1-methyl-2-vinylpyridinium trifluoromethanesulfonate (M2VP, oxidized glutathione) were precipitated with 5% (w/v) metaphosphoric acid (MPA). Liver samples were homogenized (1:10) in 15% (w/v) trichloroacetic acid (TCA) containing 1 mM EDTA for total glutathione determination and in 6% (v/v) perchloric acid (PCA) containing freshly prepared 3 mM M2VP and 1 mM EDTA for oxidized glutathione (GSSG) determination on ice. After centrifugation at 10 000 g for 10 min, 50 μl of supernatant (from deproteinized whole blood or liver homogenate) was added to 50 μL of glutathione reductase (1U) and 50 μL of 0.3 mM DTNB. The reaction was initiated by addition of 1 mM NADPH to a final volume of 200 μL. The change in absorbance was monitored at 412 nm for 3 min and levels calculated using pure GSH and GSSG as standards. Reduced glutathione (GSH) concentration was calculated as the difference between total glutathione and 2GSSG.

### Multiplex cytokine analysis

Sub samples of livers were homogenized in 10 volumes of phosphate buffered saline (10 mM PBS, pH 7.2) and centrifuged twice at 15 000 g for 15 min at 4°C. The level of four inflammatory markers TNF-α, IL-1β, IL-6 and IL-10 were determined in these homogenates using customized Milliplex™ MAP rat cytokine kits (RCYTO-80 K; Merck Millipore, St Charles, Missouri, USA), on the Bio Plex platform (Bio Plex™, Bio Rad, Laboratories, Hercules, USA) following the manufacturer’s instructions. After optimizations, liver homogenates were assayed undiluted, in a blinded manner. All analyte levels in the quality control reagents of the kits were within the expected ranges. The standard curve for all the analytes ranged from 3.2-10000 pg/ml. The analyses of the bead median fluorescence intensities were done using the Bio-Plex Manager software (version 4.1.1).

### Statistical analysis

Values were expressed as mean ± SD. Data were tested for normality and equality of variance using the Levene’s Test. Differences between groups mean were estimated using one-way analysis of variance (ANOVA) followed by the Student-Newman-Keuls test for all pairwise comparisons. The Kruskal-Wallis test, a non-parametric analogue to the one-way ANOVA was used to test for group differences when data was not normally distributed. Result were considered statistically significant at p < 0.05, or marginally significant at p < 0.1. All the statistics were carried out using MedCalc v 12.2.1 software (MedCalc software bvba, Mariakerke, Belgium).

## Results

### Phenolic content and antioxidant capacity of aqueous rooibos extract

**Figure 1:**
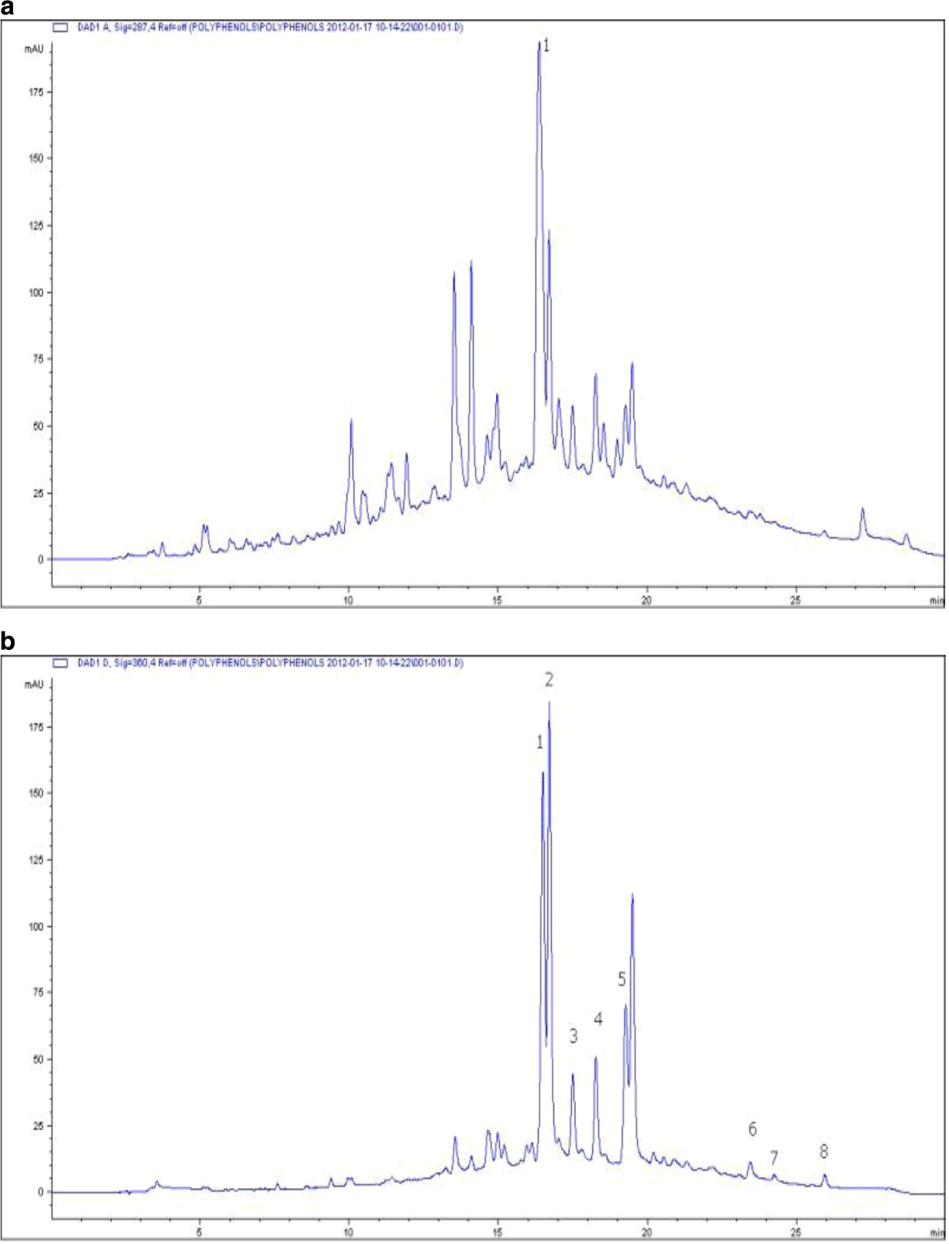
**HPLC chromatogram of fermented rooibos extract used in the study showing (a) peak 1 for aspalathin at 287 nm (b) peaks for other flavonoids.** 1, orientin; 2, iso-orientin; 3, vitexin; 4, isovitexin; 5, hyperoside/rutin; 6, quercetin; 7, luteolin; 8, chrysoeriol; at 360 nm.

Table 1 shows the results for the phenolic content and *in vitro* antioxidant capacity of the fermented rooibos extract used in the study. The total polyphenol content is 1.06 mg GAE/mL (equivalent to 0.372 mg GAE/mg soluble solids) of which the flavonols and flavanols account for 45% and 17%, respectively. The antioxidant capacity of the rooibos extract determined as ORAC, FRAP and TEAC values are also shown in Table 1. Figure 1a shows the HPLC profile of the aqueous fermented rooibos extract at 287 nm, with aspalathin (29.98 ± 0.08 μg/mL) as the most abundant flavonoid. Other major flavonoids include iso-orientin (25.98 ± 0.52 μg/mL), orientin (18.61 ± 18.61 μg/mL), hyperoside/rutin (14.55 ± 2.26 μg/mL), vitexin (6.07 ± 1.05 μg/mL), and iso-vitexin (7.18 ± 0.20 μg/mL). Minor peaks corresponding to chrysoeriol, luteolin and quercetin are also shown on the chromatogram (Figure 1b).

**Table 1:** **Phenolic content and in vitro antioxidant capacity of aqueous rooibos extract**

Aqueous rooibos extract	
Constituents	Concentration
Soluble solids (mg/mL)	2.85 ± 0.39
Total polyphenol (mg GAE/mL)	1.06 ± 0.02
Flavonol (mg QE/ mL)	0.48 ± 0.01
Flavanol (mg CE/mL)	0.19 ± 0.01
Aspalathin (μg/mL)	29.98 ± 0.08
Orientin (μg/mL)	18.61 ± 0.13
Iso-orientin (μg/mL)	25.98 ± 0.52
Vitexin (μg/mL)	6.07 ± 1.05
Iso-vitexin (μg/mL)	7.18 ± 0.20
Hyperoside/rutin (μg/mL)	14.55 ± 2.26
Quercetin (μg/mL)	0.89 ± 0.18
Luteolin (μg/mL)	0.22 ± 0.06
Chrysoeriol (μg/mL)	0.25 ± 0.01
ORAC (μmol AAE/mL)	1.69 ± 0.03
FRAP (μmol TE/mL)	5.20 ± 0.04
TEAC (μmol TE/mL)	4.79 ± 0.33

Values are mean ± SD. Soluble solid is a mean of 12 determinations while other parameters are mean of 5 determinations. AAE (ascorbic acid equivalent), CE (catechin equivalent), GAE (gallic acid equivalent), QE (quercetin equivalent), TE (trolox equivalent).

### Daily fluid and phenolic intake

The daily fluid and phenolic intake of normal and endotoxemic rats are presented in Table 2. Fluid intake, regardless of whether water or fermented rooibos was consumed, was similar across all the animal groups. Also total phenolic, flavonol and flavanol did not differ in the two groups of animals consuming the fermented rooibos.

**Table 2:** **Daily fluid and phenolic intakes of control and experimental rats**

Treatment	Water/Rooibos intake/day/100 g BW (mL)	Total phenolic intake (mg GAE/day/100 g BW)	Flavonol intake (mg QE/day/100 g BW)	Flavanol intake (mg CE/day/100 g BW)
Control	10.44 ± 1.06	ND	ND	ND
LPS	10.68 ± 0.76	ND	ND	ND
Rooibos	9.86 ± 0.88	10.45 ± 0.93	4.20 ± 0.38	1.59 ± 0.14
Rooibos + LPS	9.92 ± 0.49	10.51 ± 0.52	4.23 ± 0.21	1.60 ± 0.08

Calculations of the total phenolic, flavonol and flavanol intakes were calculated based on the soluble solid intake obtained from the average rooibos consumption per day. Values are mean ± SD (n = 10). ND (not determined), BW (body weight), CE (catechin equivalent), GAE (gallic acid equivalent), QE (quercetin equivalent), LPS (lipopolysaccharide).

### Weight parameters and serum levels of ALT, AST and LDH

The effect of rooibos consumption on body weight gain, liver weight, relative liver weight and serum levels of liver function enzymes are depicted in Table 3. Consuming the fermented rooibos extract without LPS treatment did not have any adverse effect on any of the weight parameters, as well as the serum levels of ALT, AST and LDH. No mortality was recorded in the LPS-challenged groups within the 16 hours before sacrifice. Intraperitoneal injection of LPS did not have any adverse effect on the body weight gain, liver weight or relative liver weight of the rats when compared with the control rats. There was a significant elevation (p < 0.05) in the serum levels of ALT, AST and LDH with the LPS challenge when compare to control rats, resulting in 56, 88 and 50% significant (p < 0.05) increases, respectively. Consumption of the fermented rooibos extract (with LPS challenge) significantly (p < 0.05) reduced the elevation in serum AST (21%) and LDH (28%), while serum ALT was marginally (p < 0.1) reduced by 19% when compared with rats injected with LPS only.

**Table 3:** **Effects of rooibos supplementation on weight parameters and serum marker enzymes in all experimental rats**

Treatment	Control	LPS	Rooibos	Rooibos + LPS
Weight gain (g)	89.21 ± 17.31	91.19 ± 11.57	90.01 ± 3.53	91.61 ± 6.20
Liver weight (g)	11.83 ± 0.82	12.36 ± 1.25	12.42 ± 1.25	12.64 ± 0.65
RLW (%)	3.15 ± 0.22	3.36 ± 0.43	3.27 ± 0.25	3.30 ± 0.14
ALT (U/L)	105.69 ± 28.93	165.29 ± 39.60^*^	109.31 ± 11.32	133.95 ± 39.60^##^
AST (U/L)	88.98 ± 12.53	167.22 ± 34.82^*^	82.98 ± 9.91	131.64 ± 33.92^#^
LDH (U/L)	234.92 ± 74.55	351.36 ± 81.64^*^	219.22 ± 58.16	252.67 ± 76.62^#^

Values are mean ± SD (n = 9-10). *Significantly different from control (p < 0.05). ^#^Significantly different from LPS (p < 0.05). ^##^Marginally different from LPS (p < 0.1). ALT (alanine aminotransferase), AST (aspartate aminotransferase), LDH (lactate dehydrogenase), LPS (lipopolysaccharide), RLW (relative liver weight).

### Plasma and liver antioxidant capacity and lipid peroxidation markers

The antioxidant capacity of plasma and liver homogenates were assessed as total polyphenol content and ORAC values (Table 4). LPS injection significantly (p < 0.05) reduced the plasma total polyphenol content, with rooibos supplementation able to reverse this reduction to a level comparable to that of the control. The plasma and liver ORAC of rats challenged with LPS were not significantly different (p > 0.05) from that of the control rats, regardless of whether the animals were consuming rooibos or not. Table 4 also shows the effect of the fermented rooibos extract supplementation on plasma and hepatic lipid peroxidation in normal and endotoxemic rats. Lipid peroxidation was assessed as conjugated dienes (CD) and malondialdehyde (MDA) formation. Plasma and hepatic CDs were similar across all groups of animals. MDA levels were significantly (p < 0.05) elevated in the plasma (22%) and liver (43%) of LPS rats compared with control rats. In LPS-challenged rats consuming the fermented rooibos extract, the plasma and hepatic elevation in MDA was significantly (p < 0.05) reduced by 27 and 22%, respectively.

**Table 4:** **The effect of rooibos supplementation on in vivo total antioxidant capacity and markers of lipid peroxidation in plasma and liver of all experimental rats**

Tissue	Parameter	Control	LPS	Rooibos	Rooibos + LPS
Plasma	Total polyphenol	96.69 ± 9.49	73.37 ± 6.83^*^	91.94 ± 8.49	85.69 ± 12.24^**#**^
	ORAC	1791.67 ± 167.21	1499.48 ± 375.47	1842.11 ± 345.04	1708.30 ± 528.84
	CD	85.79 ± 5.81	88.40 ± 8.56	84.34 ± 10.66	85.11 ± 8.17
	MDA	1.96 ± 0.21	2.40 ± 0.20^*^	1.79 ± 0.25	1.76 ± 0.32^**#**^
Liver	ORAC	19.06 ± 4.33	17.40 ± 4.14	21.98 ± 3.27	18.51 ± 4.50
	CD	10.38 ± 1.12	12.78 ± 0.79	11.62 ± 0.62	11.84 ± 0.47
	MDA	64.83 ± 5.46	92.65 ± 7.57^*^	64.44 ± 7.57	72.27 ± 7.79^**#**^

Values are mean ± SD (n = 9-10) *Significantly different from control (p < 0.05). ^#^Significantly different from LPS (p < 0.05). CD (conjugated dienes; nmole/L in plasma, nmole/g tissue in liver), MDA (malondialdehyde; μmole/L in plasma, μmole/g tissue in liver), LPS (lipopolysaccharide), ORAC (oxygen radical absorbance capacity; μmole trolox equivalent/L in plasma, μmole trolox equivalent/g tissue in liver), Total polyphenol (μmole gallic acid equivalent/L).

### Antioxidant enzymes and glutathione status

The effect of aqueous fermented rooibos extract consumption on antioxidant enzyme activities and glutathione redox status in the blood and liver of control and LPS-treated rats are shown in Table 5. In the erythrocytes, SOD and CAT activities were not affected by LPS injection. However, the activities of GPx and GR were significantly (p < 0.05) reduced in the LPS-treated rats when compared with the control rats. Consuming the aqueous rooibos extract marginally (p < 0.1) increased the GPx and GR activities in the LPS-challenged rats. Hepatic SOD and GPx activities were significantly (p < 0.05) decreased, while CAT activity was significantly (p < 0.05) increased as a result of the LPS challenge. Consumption of fermented rooibos extract in LPS-challenged rats significantly (p < 0.05) reversed the changes induced in hepatic SOD, CAT and GPx activities. In the blood, the LPS challenge resulted in a significantly (p < 0.05) increased GSSG level of 18%, while depleting the GSH level as well as the GSH: GSSG ratio by 34 and 39%, respectively, when compared to the control rats. Consumption of the fermented rooibos extract in LPS-challenged rats restored the GSH and GSH: GSSG ratio to levels comparable to those of the control rats, while not showing any effect on GSSG levels. Although, hepatic GSH levels were not affected by the LPS challenge in the LPS only rats when compared to the control rats, hepatic GSSG levels were significantly (p < 0.05) elevated. As a result, a significant (p < 0.05) decrease in hepatic GSH: GSSG ratio was observed in the LPS only rats when compared to the control rats. Aqueous rooibos extract consumption in the LPS treated rats significantly reduced the elevation in GSSG and improved the GSH: GSSG ratio to values similar to those found in the control rats.

**Table 5:** **The effect of rooibos supplementation on antioxidant enzymes activity and glutathione redox status in the blood and liver of all experimental rats**

Tissue	Parameter	Control	LPS	Rooibos	Rooibos + LPS
Blood	CAT	0.21 ± 0.05	0.22 ± 0.02	0.27 ± 0.04	0.20 ± 0.04
	SOD	22.35 ± 3.82	22.25 ± 3.25	25.46 ± 3.30	19.24 ± 1.72
	GR	0.15 ± 0.01	0.09 ± 0.04^*^	0.15 ± 0.03	0.12 ± 0.05^##^
	GPx	0.18 ± 0.03	0.14 ± 0.02^*^	0.23 ± 0.03	0.16 ± 0.03^##^
	GSH	906.11 ± 173.54	596.04 ± 135.59^*^	905.09 ± 135.20	862.60 ± 112.25^#^
	GSSG	173.47 ± 34.25	205.29 ± 8.69^*^	185.45 ± 35.99	202.92 ± 17.52
	GSH:GSSG	4.76 ± 1.22	2.90 ± 0.65^*^	5.18 ± 1.86	4.27 ± 0.59^#^
Liver	CAT	0.11 ± 0.01	0.23 ± 0.03^*^	0.11 ± 0.03	0.16 ± 0.03^#^
	SOD	55.01 ± 5.27	42.06 ± 6.26^*^	61.50 ± 3.85	57.62 ± 6.92^#^
	GR	3.99 ± 1.12	2.69 ± 0.62	3.80 ± 0.80	3.37 ± 0.79
	GPx	0.16 ± 0.02	0.12 ± 0.02^*^	0.16 ± 0.01	0.15 ± 0.01^#^
	GSH	6.38 ± 0.58	6.37 ± 0.83	7.29 ± 0.96	7.41 ± 0.75
	GSSG	0.32 ± 0.10	0.46 ± 0.09^*^	0.28 ± 0.04	0.35 ± 0.08^#^
	GSH:GSSG	21.42 ± 6.32	13.64 ± 2.39^*^	25.64 ± 4.66	21.98 ± 5.34^#^

Values in columns are mean ± SD (n = 9-10). *Significantly different from control (P < 0.05). ^#^Significantly different from LPS (p < 0.05). ^##^Marginally different from LPS (p < 0.1). CAT (catalase, μmole H_2_O_2_ consumed/min/μg protein), GR (glutathione reductase, μmole NADPH oxidized/min/μg protein), SOD (superoxide dismutase, U/mg protein), GPx (glutathione peroxidase, nmole NADPH oxidized/min/μg protein). GSH (reduced glutathione, μmole/L in blood, μmole/g tissue in liver), GSSG (oxidized glutathione, μmole/L in blood, μmole/g tissue in liver), LPS (lipopolysaccharide).

### Hepatic cytokine levels

**Figure 2:**
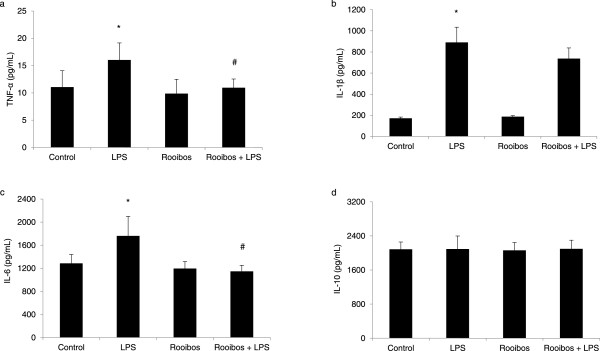
**Effect of aqueous fermented rooibos extract supplementation on hepatic (a) TNF-α (b) IL-1β (c) IL-6 and (d) IL-10 levels innormal and endotoxemic rats.** Bars represent mean ± SD (n = 8-10). ^*^Significantly different from control at p < 0.05. ^#^Significantly different from LPS at p < 0.05. LPS (lipopolysaccharide).

Figure 2 shows the effect of the aqueous fermented rooibos extract consumption on hepatic cytokine levels in normal and LPS-treated rats. A significant (p < 0.05) increase in the levels of TNF-α (45%), IL-1β (419%) and IL-6 (37%) (Figures 2a, 2b and 2c, respectively) were observed in the liver of LPS-treated rats compared to control rats. Consuming the fermented rooibos extract significantly (p < 0.05) reduced the LPS-induced elevation in TNF-α and IL-6 to levels comparable to those observed in the negative control rats, however, no effect was seen in the IL-1β levels of LPS rats that consumed the rooibos extracts. The hepatic levels of the anti-inflammatory cytokine IL-10 (Figure 2d) was similar across all treatment groups, including the rooibos as well as the LPS and control groups.

## Discussion

The focus of this study was to investigate the acute effects of LPS-induced hepatic oxidative stress and inflammatory responses, and the possible protection offered by oral administration of an aqueous fermented rooibos extract. The liver plays an important and central role in the regulation of entry and metabolism of LPS upon exposure. Mostly, the hepatic and systemic toxicities of LPS have been attributed to the release of chemical mediators such as superoxide, nitric oxide and pro-inflammatory cytokines, including TNF-α, IL-1β and IL-6, which are all formed as a result of the binding of LPS to the CD14/LPS-binding protein and Toll-like receptor-4 (TLR4) on the surface of Kupffer cells [3, 32]. Oxidative stress is a well-known mechanism of LPS-induced hepatic injury, and the redox imbalance produced may result in depletion of endogenous antioxidants such as the antioxidant enzymes and alteration of GSH redox status. Thus, augmenting the antioxidant defense system becomes necessary, especially during infections or periods of chronic oxidative insult. Whole extracts or isolated compounds from plants are popular applications to reverse and/or prevent hepatotoxicity and oxidative stress produced by noxious agents, such as LPS and these beneficial effects may be attributed to their antioxidant and anti-inflammatory properties.

In this study, it was observed that a single injection of LPS did not have a negative effect on the body weight gain, absolute liver weight and relative liver weight of the rats. However, LPS injection resulted in hepatic injury as indicated by an elevation in the levels of serum ALT, AST, and LDH, all circulating markers of hepatocyte injury. The hepatic function marker enzymes are cytoplasmic in nature but are usually leaked into circulation when liver damage occurs due to an alteration in membrane permeability. This observation is in accordance with several earlier reports which have shown that LPS induces hepatic damage, and as a consequence, increases the level of serum aminotransferases [33–35]. Results from the current study showed that supplementation with the aqueous fermented rooibos extract for 4 weeks prior to the LPS challenge, reversed the induced damage in the liver. Previous reports have demonstrated the ability of some polyphenol-rich plant extracts such as *Artemisia absinthium* [36], *Salvia plebeia* [37], *Eucalyptus globulus* [38] and *Hibiscus sabdariffa* [39] in ameliorating LPS-induced hepatic injury. The protective effect of rooibos extract observed in our study may be due to the ability of the flavonoids in rooibos to stabilize and maintain the integrity of the hepatocyte membrane, as well as repair damaged hepatic tissues by stimulating hepatocyte regeneration and hepatocellular protein synthesis.

Results from a previous study have shown activation of Kupffer cells during endotoxemic episodes to result in the secretion of a wide variety of cytokines, including TNF-α, IL-1β and IL-6 [40]. Up-regulation of cytokine production during LPS-induced endotoxemia is a well-known phenomenon, and evidence has shown that increased levels of pro-inflammatory cytokines from neutrophils in the liver were associated with liver cell damage [41]. Tumor necrosis factor-α, and IL-6 are two key cytokines involved in tissue damage during sepsis, although it has been suggested that TNF-α is the central mediator regulating other subsequent events [42]. In the present study, LPS significantly increased the level of hepatic TNF-α, IL-1β and IL-6. Feeding rooibos for 4 weeks prior to the LPS challenge was able to reverse the increase in TNF-α and IL-6 observed in the liver to a level that was comparable to what was seen in untreated control animals. However, no reversal was seen in the level of IL-1β with rooibos extract supplementation. Although Hendricks & Pool [13], in an *in vitro* study reported that rooibos tea induced a higher IL-6 and a lower IL-10 when added to endotoxin-stimulated white blood cells, literature supporting the inhibition of cytokines by rooibos is scarce. Thus, we are tempted to speculate that the protective effect shown by rooibos on cytokine formation in this study may be due to the plethora of antioxidant phytochemicals found in rooibos, since previous reports have shown that flavonoids found in rooibos such as luteolin, rutin and quercetin reduced LPS-induced expression of cytokines [43, 44]. We also propose that the mechanism for the observed reduction in hepatic TNF-α and IL-6 may involve the ability of antioxidant phytochemicals present in rooibos to inhibit the activation of Kupffer cells by LPS, leading to inhibition of TNF-α synthesis and subsequently synthesis of IL-6. Though the level of nuclear factor- κB (NF-κB) was not measured in this study, given its central role in signaling and the inflammatory cascade, it is proposed that inhibiting the activation of NF-κB by rooibos, may be an additional mechanism for the observed decrease in hepatic TNF-α and IL-6 level. IL-10 is an anti-inflammatory cytokine produced by monocytes and lymphocytes, while previous reports have indicated that it is antagonistic to TNF-α in its modulation of the inflammatory response [45]. IL-10 has been reported to down-regulate TNF-α, as well as other cytokine production, by suppressing their gene expression in an autocrine-like feedback loop [46]. However in this study, we observed that the level of IL-10 was similar across all control and LPS-treated groups, indicating that the inhibition of TNF-α formation observed with rooibos extract supplementation may be independent of the activation of the feedback loop of IL-10.

Nitric oxide (NO) and superoxide anion (O_2_^•-^) are two free radicals secreted during activation of Kupffer cells under LPS insult. NO reacts with O_2_^•-^ to form peroxynitrite which is a potent cytotoxic, oxidative agent that can elicit lipid peroxidation [47]. LPS-induced lipid peroxidation is an index of oxidative stress, and several previous studies have reported enhanced lipid peroxidation in many tissues (including liver, heart, brain, small intestine and stomach) of rats [2, 33–35, 48]. Under conditions of oxidative stress, reactive oxygen and nitrogen species (RONS) attack the polyunsaturated fatty acids (PUFAs) of cell membranes causing destabilization, disintegration and alteration in membrane fluidity and permeability, all events which increase the rate of protein degradation and eventually leads to cell lysis [40]. Decomposition products of lipid hydroperoxides such as MDA and 4-HNE can also cause chaotic cross-linkage with protein and nucleic acids, leading to oxidative protein and DNA damage [49]. In this study, plasma and hepatic CD and MDA, as markers of lipid peroxidation, were measured. While plasma and hepatic CD were unaffected by LPS treatment, elevated levels of plasma and hepatic MDA were observed. Pre-feeding rooibos extract for 4 weeks in the LPS-challenged rats, inhibited the formation of MDA in the plasma and liver. A large number of *in vitro* studies have established the excellent free radical scavenging ability of rooibos, and this has been confirmed in many *in vivo* studies. The ability of rooibos to lower lipid peroxidation and modulate oxidative stress has been demonstrated in rat brain [50], liver [9, 51, 52], as well as in humans at risk [10, 11]. Since rooibos is a polyphenol-rich herbal tea, its polyphenolic compounds may be able to bind RONS directly and scavenge them or act as sacrificial antioxidants to inhibit the lipid peroxidation cascade as seen in this study.

The impairment of the antioxidant defense system is a critical step in LPS-induced hepatic injury. Evidence has shown that a LPS insult is characterized by change in tissue and circulating antioxidant enzymes levels, as well as non-enzymatic antioxidants, including GSH [33, 34, 40]. In our study, plasma SOD and CAT were unaffected by LPS treatment, while activities of GPx and GR were significantly reduced. In the liver, the activity of SOD was inhibited by LPS treatment. This inhibition is not surprising since the O_2_^•-^ has been implicated as one of the toxic mediators responsible for most toxicities observed in LPS-induced cellular injury, and SOD, a metalloprotein, is a key enzyme involved in the protection of cells by spontaneously dismutating O_2_^•-^ to H_2_O_2_. The H_2_O_2_ produced by SOD is usually decomposed to water and oxygen by the hemoprotein CAT, localized in the peroxisomes. LPS treatment significantly increased the activity of hepatic CAT in this study. An increase that may be an adaptive physiological response to overproduction of H_2_O_2_ resulting from SOD action. GPx and GR are important enzymes of the glutathione defense system. While GPx catalyses the reduction of H_2_O_2_ and lipid hydroperoxides using GSH as a co-substrate, GR regenerates GSH from GSSG at the expense of NADPH. In this study, the activities of both GPx and GR were significantly depleted by LPS treatment, an indication of their inactivation and failure of the antioxidant enzymes to overcome the influx of RONS after LPS exposure [53]. Results from this study further showed that feeding rooibos for 4 weeks prior to the LPS challenge reversed the changes observed in the activities of SOD, CAT, GPx and GR in the liver. The modulation of the antioxidant enzymes activities observed in the LPS-challenged rats consuming rooibos could be ascribed to the direct quenching of RONS generated by LPS, since antioxidant components of rooibos are established free radical scavengers. Furthermore, the up-regulation and/or down-regulation of the gene expression of the antioxidant enzymes may be an additional mechanism that should be elucidated in future studies. Reduced glutathione (GSH) is the major non-protein thiol in plant and animal cells. It is essential for the regulation of a variety of cellular functions, playing an important role in intracellular protection against ROS and other free radicals [54]. Because of its sulphydryl (–SH) group, it can function as a nucleophile, forming conjugates with many xenobiotics and/or their metabolites and also serve as a reductant in the metabolism of hydrogen peroxide and other organic peroxides [55]. During interaction with free radicals, the –SH group of GSH becomes oxidized, leading to the formation of corresponding disulfide compound (GSSG). Thus, a depletion of GSH is usually associated with an increase in GSSG concentration and a lowered GSH:GSSG redox ratio during conditions of oxidative stress [56, 57]. Results from the current study revealed a decrease in GSH (blood) and an increase in GSSG (liver) in rats challenged with LPS. These events invariably resulted in a decrease in the GSH:GSSG ratio in both tissues of LPS-challenged rats. Feeding rooibos to LPS-treated rats, restored the GSH:GSSG ratio to values comparable to those found in the negative control animals, indicating that rooibos extract is able to protect against LPS-induced glutathione imbalance. The protection shown by rooibos in this study is in agreement with previous studies where rooibos has been reported to improve the GSH:GSSG ratio in rat hearts subjected to ischaemia/reperfusion injury [58], rats oxidatively challenged with *t*-BHP [9] and human populations at the risk for cardiovascular diseases [11]. The ability to improve the GSH: GSSG ratio via increasing GSH (plasma) and/or decreasing GSSG (liver), shown by rooibos can be ascribed to the ability of their individual antioxidant components to quench free radicals and up-regulate the synthesis of GSH as suggested in some previous studies [59].

It is important to point out that since this study was conducted in male rats, it is possible that the results obtained may or may not be applicable to female rats. Several reports have shown gender-related differences in liver damage after various types of insults [60, 61]. Some studies in animals reported a sex-related difference to oxidative stress and activities of cellular antioxidant enzymes which may be organ specific [62–64]. These studies showed that female animals may be less susceptible to oxidative stress-induced liver injury compared to male animals. Though gender-related differences in susceptibility to xenobiotic-induced liver injury is controversial and its specific mechanism(s) is still not fully understood, differences in circulating sex hormones, hepatic expression of sex hormone receptors and pattern of growth hormone secretion have all been mentioned to be responsible [61].

## Conclusion

This study provides the first *in vivo* evidence of an anti-inflammatory effect of rooibos in LPS-induced hepatic injury in rats. LPS-induced cytokine secretion has been shown to require the production of reactive oxygen and nitrogen species, resulting in lipid peroxidation as demonstrated in this study. Results from this study further demonstrates that rooibos is able to suppress LPS-triggered oxidative stress and inflammatory responses in the liver by attenuating liver damage, lipid peroxidation, redox (GSH:GSSG) imbalance and pro-inflammatory cytokine secretion in a Wistar rat model. Rooibos contain important phytochemical constituents with excellent antioxidant properties which may in part, explain this observed anti-inflammatory activity. This suggest that rooibos may be of benefit in the prophylactic management of LPS-induced liver injury, however, future studies are necessary to fully examine the specific mechanisms underlying the protective effects shown by this herbal tea.

## Abbreviations

ALT: Alanine aminotransferase
ANOVA: Analysis of variance
AST: Aspartate aminotransferase
CAT: Catalase
CD: Conjugated dienes
DMACA: 4-(dimethylamino)-cinnamaldehyde
DTNB: 5,5’-dithiobis-2-nitrobenzoic acid
FRAP: Ferric ion reducing antioxidant power
GPx: Glutathione peroxidase
GR: Glutathione reductase
GSH: Reduced glutathione
GSSG: Oxidized glutathione
HPLC-DAD: High-performance liquid chromatography with diode array detection
IL: Interleukin
LDH: Lactate dehydrogenase
LPS: Lipopolysaccharide
MDA: Malondialdehyde, MPA, metaphosphoric acid
M2VP: 1-methyl-2-vinylpyridinium trifluoromethanesulfonate
NADPH: β-nicotinamide adenine dinucleotide phosphate reduced tetrasodium salt
NF-κB: Nuclear factor- κB
ORAC: Oxygen radical absorbance capacity
PCA: Perchloric acid
PUFA: Roly-unsaturated fatty acids
RONS: Reactive oxygen and nitrogen species
SOD: Superoxide dismutase
TBA: 2-thiobarbituric acid
TEAC: Trolox equivalent antioxidant capacity
TLR4: Toll-like receptor 4
TNF-α: Tumour necrosis factor- α.
